# Activity ex vivo of cytotoxic drugs in patient samples of peritoneal carcinomatosis with special focus on colorectal cancer

**DOI:** 10.1186/1471-2407-13-435

**Published:** 2013-09-24

**Authors:** Peter H Cashin, Haile Mahteme, Wilhelm Graf, Henning Karlsson, Rolf Larsson, Peter Nygren

**Affiliations:** 1 Department of Surgical Sciences, Section of Surgery, Akademiska Sjukhuset, Uppsala University, Uppsala S-751 85, Sweden; 2 Department of Medical Sciences, Section of Clinical Pharmacology, Akademiska Sjukhuset, Uppsala University, Uppsala S-751 85, Sweden; 3 Department of Radiology, Oncology, and Radiation Sciences, Section of Oncology, Akademiska Sjukhuset, Uppsala University, Uppsala S-751 85, Sweden

**Keywords:** Chemotherapy resistance, Cytoreductive surgery, Drug sensitivity, Fluorometric microculture cytotoxicity assay, Hyperthermic intraperitoneal chemotherapy, Peritoneal carcinomatosis

## Abstract

**Background:**

The optimal choice of cytotoxic drugs for intraperitoneal chemotherapy (IPC) in conjunction with cytoreductive surgery (CRS) for treatment of peritoneal carcinomatosis (PC) is poorly defined. We investigated drug sensitivity ex vivo in patient samples of various PC tumor types and correlated clinical outcome to drug sensitivity within the subset of PC from colorectal cancer (CRC).

**Methods:**

PC tissue samples (*n* = 174) from mesothelioma, pseudomyxoma peritonei (PMP), ovarian cancer, CRC or appendix cancer were analyzed ex vivo for sensitivity to oxaliplatin, cisplatin, mitomycin C, melphalan, irinotecan, docetaxel, doxorubicin and 5-FU. Clinicopathological variables and outcome data were collected for the CRC subset.

**Results:**

Mesothelioma and ovarian cancer were generally more drug sensitive than CRC, appendix cancer and PMP. Oxaliplatin showed the most favorable ratio between achievable IPC concentration and ex vivo drug sensitivity. Drug sensitivity in CRC varied considerably between individual samples. Ex vivo drug sensitivity did not obviously correlate to time-to-progression (TTP) in individual patients.

**Conclusions:**

Drug-sensitivity varies considerably between PC diagnoses and individual patients arguing for individualized therapy in IPC rather than standard diagnosis-specific therapy. However, in the current paradigm of treatment according to diagnosis, oxaliplatin is seemingly the preferred drug for IPC from a drug sensitivity and concentration perspective. In the CRC subset, analysis of correlation between ex vivo drug sensitivity and TTP was inconclusive due to the heterogeneous nature of the data.

## Background

Peritoneal carcinomatosis (PC) was previously regarded as an incurable form of malignant disease with a poor prognosis, and the intention of treatment was palliative. However, aggressive cytoreductive surgery (CRS) followed by intraperitoneal chemotherapy (IPC), may produce prolonged long-term survival and even cure [1-5]. The most recent development in the management of PC is the intraoperative use of hyperthermic intraperitoneal chemotherapy (HIPEC) [6].

The individual therapeutic impact of cytoreductive surgery and IPC, respectively, has not been sufficiently clarified [7]. In ovarian cancer, IPC is more active than the corresponding drug given iv and in gastric cancer, IPC has been shown to add benefit to CRS and systemic chemotherapy [8,9]. In PC from colorectal cancer (CRC), the role of IPC when added to CRS has not been elucidated in randomized trials but cytoreductive surgery, IPC, and systemic chemotherapy is favorable compared with systemic chemotherapy and palliative surgery [10]. An experimental study in the rat demonstrated a significant increase in survival when adding IPC to cytoreductive surgery vs. cytoreductive surgery alone [11].

The selection of drugs for IPC has mostly been based on the experience from systemic administration, pharmacodynamic properties, hyperthermic enhancement, technical feasibility, pharmacokinetics and tolerance [12,13]. The IPC protocols in use do not take into consideration possible differences in drug sensitivity in the different tumor types or differences in tumor cell sensitivity between individual patients. Currently, cisplatin, doxorubicin, mitomycin C, oxaliplatin and irinotecan, as single drugs or sometimes combined, are the most commonly used drugs for HIPEC treatment [14-18]. A more differential approach to drug selection for the IPC in PC might provide more benefit from this part of the treatment of PC.

With this background, we investigated ex vivo the activity of standard cytotoxic drugs in IPC on tumor cells derived from patients with various types of PC using an ex vivo model reflecting clinical drug activity. The aims were to investigate differences in drug sensitivity between various PC tumor types and individual patient samples and to investigate if differences in clinical outcome are associated with drug sensitivity within the subset of CRC.

## Methods

### Tumor sampling and cell preparation

Tumor sampling of patients with PC from appendix cancer, CRC, pseudomyxoma peritonei (PMP), ovarian cancer, or mesothelioma was performed intraoperatively during cytoreductive surgery prior to IPC. Leukemia sampling was by vein puncture at routine blood sampling and mononuclear cells (MNCs) were prepared from buffy coats from healthy blood donors. Tumour sampling and data collection was based on patient informed consent as approved by the regional ethical committee in Uppsala (Uppsala Etiknämnd: Dnr 2007/237).

Tumor cells from solid tumor tissue were prepared by collagenase digestion as described [19]. Leukemia cells and MNCs were collected by Ficoll-Hypaque (Pharmacia, Uppsala, Sweden) gradient centrifugation [20].

The cells obtained from the solid tumors were single cells or small cell clusters with ≥ 90% viability and with less than 30% contaminating non-malignant cells, as judged by morphological examinations of May-Grünwald-Giemsa-stained cytocentrifugate preparations. Approximately 85% of all samples obtained fulfilled the criteria for a successful assay (see below) and were included in this study. The numbers and types of samples included are detailed in Table 1.

**Table 1:** Number of patient samples included in the analyses

**CELL TYPE**	**TREATED ( *n *)**	**UNTREATED ( *n *)**	**TOTAL ( *n *)**
Pseudomyxoma	24	48	72
Mesothelioma	7	8	15
Appendix	11	5	16
Colorectal	39	13	52
Ovarian	15	4	19
AML	6	6	12
CLL	0	12	12
MNC	NA	NA	44

Treated/untreated refers to whether the patients sampled had previously received chemotherapy (treated) or not (untreated).

*Abbreviations*: *AML* acute myeloblastic leukemia, *CLL* chronic lymphocytic leukemia, *MNC* mononuclear cells, *NA* not applicable.

### Drugs and measurement of drug sensitivity ex vivo

The cytotoxic drugs melphalan (Mel; GlaxoSmithKline, Stockholm, Sweden), cisplatin (Cisp; Bristol-Myers Squibb, Stockholm, Sweden), oxaliplatin (Oxali; Sanofi-Synthelabo, Stockholm, Sweden), doxorubicin (Dox; Pfizer, Stockholm, Sweden), docetaxel (Doce), 5-fluorouracil (5FU; Roche, Stockholm, Sweden), mitomycin C (MitC; Bristol-Myers Squibb) and irinotecan (Irino; Pfizer) were from commercially available clinical preparations. The drugs were tested at three 10-fold dilutions from the maximal concentration (μM) of 100 for Mel, 100 for Cisp, 100 for Oxali, 10 for Dox, 100 for Doce, 1000 for 5-FU, 100 for MitC and 1000 for Irino. Irino shows relevant activity ex vivo under conditions of the FMCA despite being considered as a prodrug [21].

The semi-automated fluorometric microculture cytotoxicity assay (FMCA), described in detail previously, was used to assess drug sensitivity [22]. The method is based on measurement of fluorescence generated from hydrolysis of fluorescein diacetate (FDA) to fluorescein by cells with intact plasma membranes. 384-well microplates (Nunc) were prepared with 5 μl drug solution at 10 times the final drug concentration using the pipetting robot BioMek 2000 (Beckman Coulter). The plates were then stored at -70°C until further use.

Tumor cells from patient samples (5,000 cells/well for the PC samples and 40,000 cells per well for leukemia and MNCs) in 45 μl were seeded in the drug-prepared 384-well plates using the pipetting robot Precision 2000 (Bio-Tek Instruments Inc., Winooski, VT). Three columns without drugs served as controls and one column with medium only served as blank.

The culture plates were then incubated at 37°C in humidified atmosphere containing 95% air and 5% CO_2_. After 72 h incubation, the culture medium was washed away and 50 μl/well of a physiological buffer containing 10 μg/ml of the vital dye fluorescein diacetate (FDA) were added to control, experimental and blank wells. After incubation for 30–45 min at 37°C, the fluorescence from each well was read in a Fluoroscan 2 (Labsystems OY, Helsinki, Finland).

### Quality control and quantification of results

Quality criteria for a successful assay were: ≥ 70% tumor cells in the cell preparation prior to incubation and/or on the assay day, a fluorescence signal in control cultures of ≥ five times mean blank values, and a coefficient of variation of cell survival in control cultures of ≤ 30%. The results obtained by the viability indicator FDA are presented as survival index (SI), defined as the fluorescence of the test expressed as a percentage of control cultures, with blank values subtracted.

Concentration-response SI data were used to calculate the 50% inhibitory concentrations, i.e. the drug concentration producing a SI of 50%, (IC_50_). This was done using non-linear regression to a standard sigmoidal dose–response model in GraphPad Prism version 5 for Mac (GraphPad Software, San Diego, CA, USA). Data are presented as mean values ± SE for the number of experiments/samples indicated.

### Subgroup analysis of patients with peritoneal carcinomatosis of colorectal origin

Histopathological data concerning lymph node positive status, tumor grading, mucinous status, and signet cells were collected from the pathological report of the primary tumor resection. Date and cause of death, time to disease progression, completeness of cytoreduction score (CC), systemic chemotherapy prior to cytoreductive surgery and use of adjuvant systemic chemotherapy after CRS and IPC were also collected. Time to progression (TTP) was selected as the clinically most relevant outcome measure when investigating the correlation between ex vivo drug sensitivity and clinical outcome.

Out of the 52 patients with PC from CRC, fifteen did not receive IPC due to too advanced disease. The IPC in the remaining 37 patients was intraoperative hyperthermic IPC (HIPEC) or sequential postoperative intraperitoneal chemotherapy (SPIC). HIPEC consisted of oxaliplatin (360–460 mg/m^2^) administered intraperitoneally (ip) with 5-FU (400–500 mg/m^2^ plus folic acid) intravenously (iv) in 12 patients. HIPEC with irinotecan and oxaliplatin (360 mg/m^2^ of each drug) with 5-FU and folic acid iv was used in 18 patients. SPIC with 5-FU (500 mg/m^2^) infusion ip through a PORT á CATH (No. 21-2000-04, SIMS Deltec, Inc., St Paul, MN, USA) at 4–6 week intervals for 6 months postoperatively was used in 7 patients. For systemic chemotherapy prior to the IPC (n = 39), the interval between last administration and surgery had to be at least 4 weeks. Adjuvant chemotherapy was administered in 15 patients and the most common regimen was oxaliplatin and 5-FU (n = 8).

There were 27 patients with a CC score of 0 receiving IPC treatment and these were considered suitable for assessment of the ex vivo – in vivo correlation. There was missing data on recurrences in 4 patients and so the analysis was possible in 23 patients. In line with previous principles for correlation of ex vivo drug sensitivity to clinical outcome [19], patients were scored as ex vivo 'sensitive’ if they were treated with at least one drug with IC_50_ value below the median for the whole study cohort and as ex vivo 'resistant" if they had received only drugs with all IC_50_ values above the median.

### Statistics

Statistical inferences between several means were performed by one-way ANOVA with Tukey’s multiple comparison post-test of group means or, for comparison of two means, by Student’s t-test. Cross-resistance between selected drugs was analyzed by Spearman rank correlation. The slope of the regression line was calculated with the least squares method. Time to progression was compared using the Kaplan-Meier curve’s median survival and the log-rank test. For the CRC subgroup, the prognostic importance of clinicopathological variables and ex vivo drug sensitivity for TTP was assessed in a Cox regression model. All univariate results with p < 0.05 were included in the multivariable analysis using an all-effects function of both forward and backward stepwise analysis. The level of significance for all statistical tests was set to p < 0.05. The statistics software used in these analyses was STATISTICA 10.1 (StatSoft Inc, Tulsa, OK, USA).

## Results

### Patient samples

In total, 174 tumor samples from patients operated on for PC, as detailed in Table 1, fulfilled the quality criteria and were analyzed for drug sensitivity. The majority of patients had PMP or CRC and the majority of samples were from patients previously treated with chemotherapy. The clinical characteristics for the CRC patient subgroup are detailed in Table 2.

**Table 2:** Clinical characteristics of the CRC subgroup of 52 patients

**Characteristics (n, except for age in years and PCI in score units)**	**Colorectal cancer**
Age – mean years (95% CI)	55 (51–59)
Gender – Male/Female	21/31
IPC administered	37
SPIC	7
HIPEC	30
Oxaliplatin	12
Oxaliplatin + irinotecan	18
PCI – mean (95% CI)	21 (18–24)
CC score	
0	27
1	5
2	4
3	16
Diagnosis	
Colon	47
Rectum	5
Preoperative chemo	39
Oxaliplatin/5-FU ± bev	30
Oxaliplatin/5-FU + cet	1
Oxaliplatin/capecitabine ± bev	4
Irinotecan/5-FU + bev	1
Capecitabine	1
5-FU alone	2
Adjuvant systemic chemo	15
Oxaliplatin/5-FU ± bev	8
Irinotecan/5-FU ± bev	2
Capecitabine	1
5-FU alone	1
Missing data on drug	3

Abbreviations: *CRC* colorectal cancer, *PCI* peritoneal cancer index, *IPC* intraperitoneal chemotherapy, *SPIC* sequential postoperative intraperitoneal chemotherapy, *HIPEC* hyperthermic intraperitoneal chemotherapy, *CC* completeness of cytoreduction, *Chemo* chemotherapy, *Bev* bevacizumab, *Cet* cetuximab.

### Drug sensitivity ex vivo

**Figure 1:**
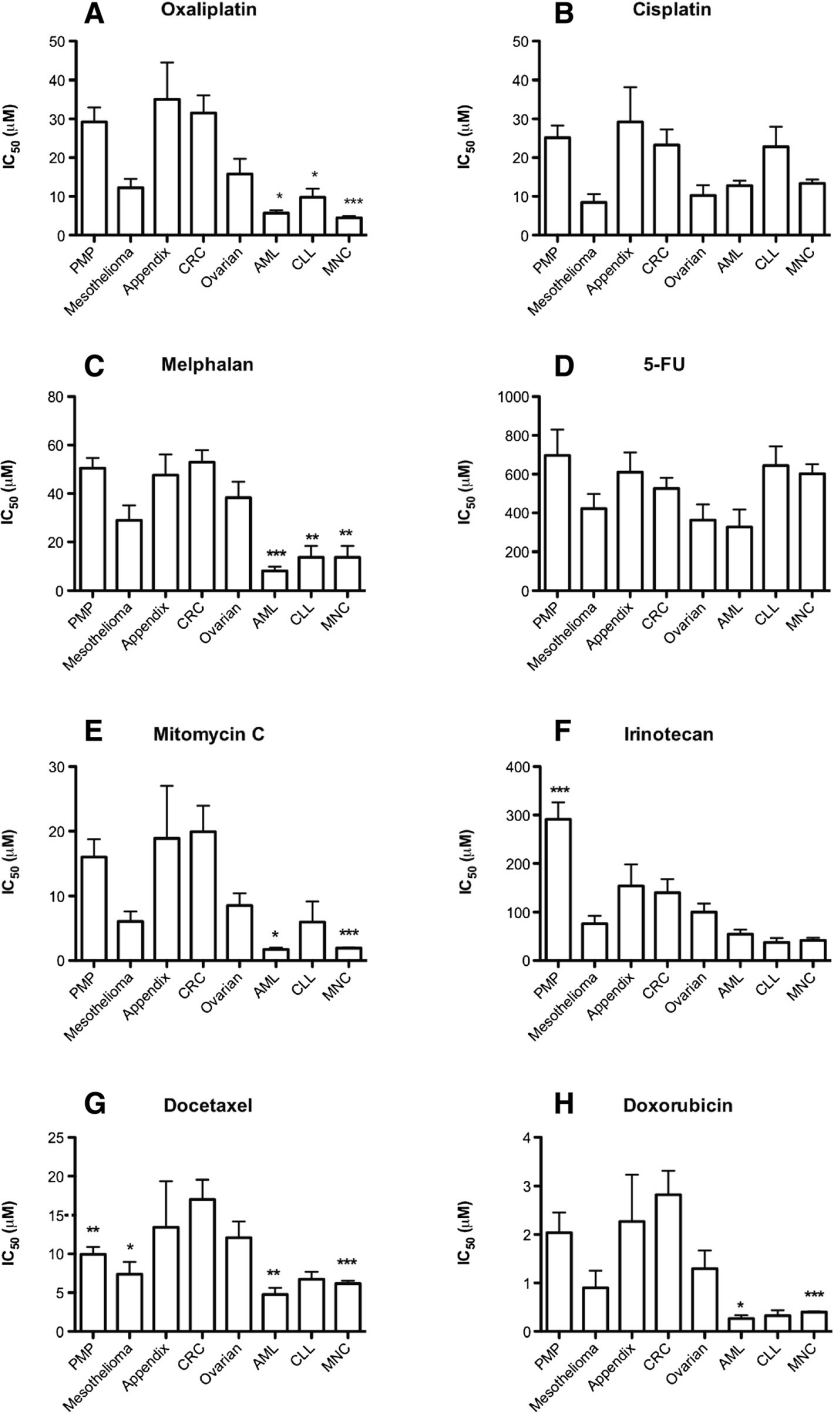
**IC**_**50 **_**values for standard drugs in all peritoneal carcinoma samples investigated divided for the subtypes indicated.** Results are presented as means values + SE. Statistical inference was calculated with 1-way ANOVA with Dunnet’s post-test and with the colorectal cancer samples as reference. The following panel of 8 drugs were investigated: **A** – Oxaliplatin, **B** – Cisplatin, **C** – Melphalan, **D** – 5FU, **E** – Mitomycin C, **F** – Irinotecan, **G** – Docetaxel, **H** – Doxorubicin. *, ** and *** denotes *P* < 0.05, 0.001 and 0.0001 vs. colorectal samples, respectively. Absence of asterixes means that no statistical differences compared to colorectal samples were observed.

Samples from CRC, appendix cancer, and PMP generally had higher IC_50_ values compared to the other groups with the exception of 5-FU (Figure 1). Mesothelioma samples were surprisingly sensitive across the panel of drugs similarly to ovarian cancer. As expected the leukemia samples were mostly more sensitive or as sensitive as the mesothelioma and ovarian cancer samples.

Based on the IC_50_ values and the ip concentrations of each drug reached during IPC, as reported in the literature, the ratios between the ip concentrations and the IC_50_ values were calculated for each drug and PC diagnosis (Table 3). High ratios would in theory be most beneficial. Across all diagnoses, the clearly most beneficial ratio was observed for oxaliplatin.

**Table 3:** Concentration ratios between IPC C_max_ and ex vivo IC_50_ values

**C**_**max**_^**†**^	**IC**_**50**_	**C**_**max**_	**IC**_**50**_	**C**_**max**_	**IC**_**50**_	**C**_**max**_	**IC**_**50**_	**C**_**max**_	**IC**_**50**_	**C**_**max**_
	**CRC**	**/IC**_**50**_	**Meso**	**/IC**_**50**_	**PMP**	**/IC**_**50**_	**App**	**/IC**_**50**_	**Ova**	**/IC**_**50**_
	**μM**	**CRC**	**μM**	**Meso**	**μM**	**PMP**	**μM**	**App**	**μM**	**Ova**
Oxa [23]										
573 μM^‡^	31	18.5	12	47.7	29	19.9	35	16.4	15	38.2
Cis [24]										
92 μM^‡^	23	4.0	8	11.5	25	3.7	30	3.1	9	10.2
MMC [25]										
30 μM^‡^	20	1.5	5	6	16	1.9	19	1.6	8	3.7
Iri [17]										
197 μM^‡^	139	1.4	75	2.6	290	0.7	150	1.3	100	2.0
Dox [14]										
17 μM^‡^	2.8	6.1	0.9	19.1	2.0	8.6	2.3	7.5	1.2	14.3

^†^ Maximum concentration achieved at the beginning of the IPC treatment.

^‡^ Dosage during HIPEC: oxa - 460 mg/m^2^, cis - 50 mg/m^2^, MMC - 35 mg/m^2^, Iri – 350 mg/m^2^, Dox – 15 mg/m^2^.

*Abbreviations*: *IPC* intraperitoneal chemotherapy, *CRC* colorectal cancer, *Meso* mesothelioma, *PMP* pseudomyxoma peritonei, *App* appendix cancer, *Ova* ovarian cancer, *Oxa* oxaliplatin, *Cis* cisplatin, *MMC* mitomycin C, *Iri* irinotecan, *Dox* doxorubicin, *N/A* not available, *HIPEC* hyperthermic intraperitoneal chemotherapy.

**Figure 2:**
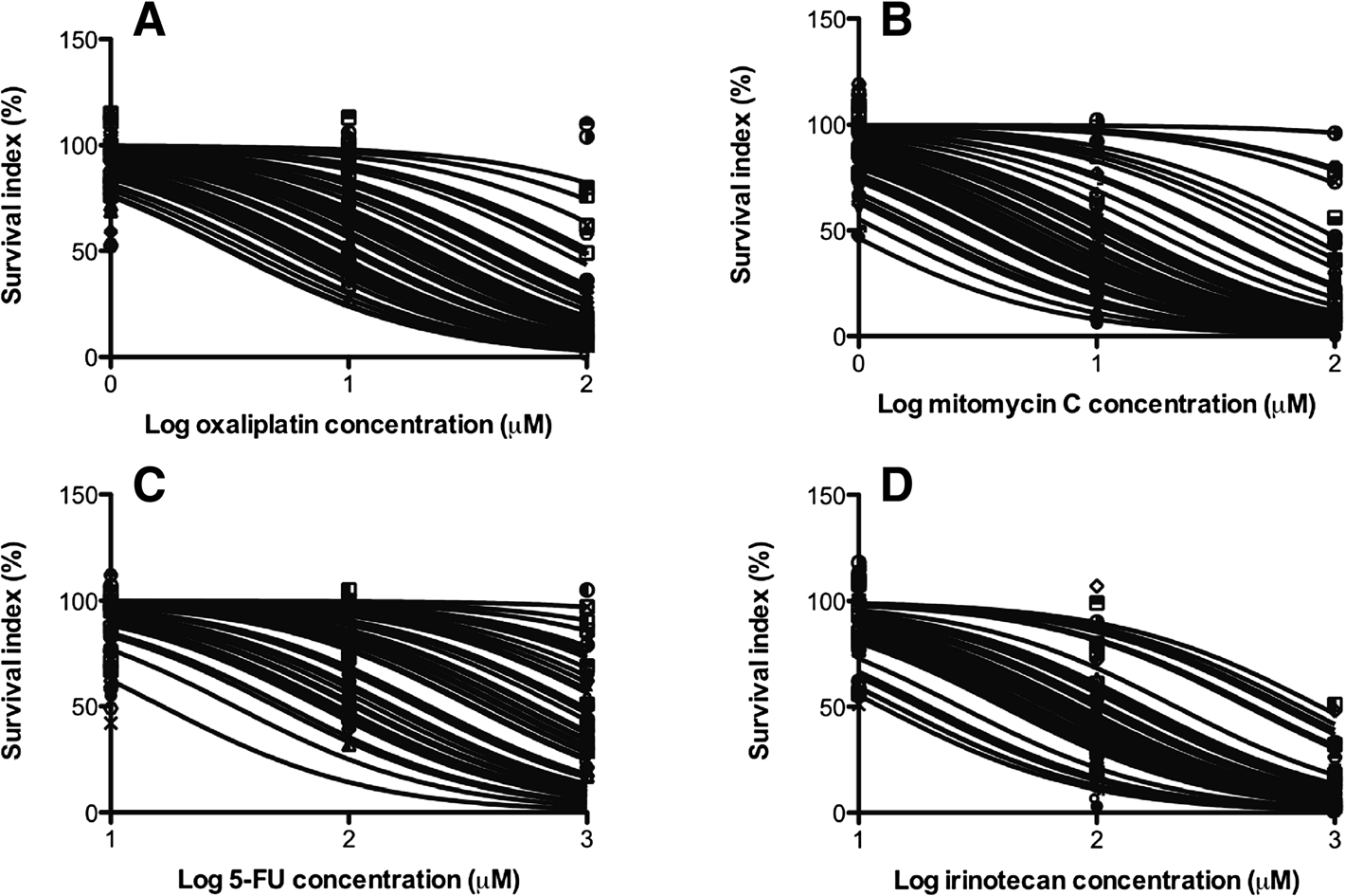
**Tumor cell sensitivity, expressed as survival index (SI%) of the colorectal cancer samples for the indicated standard cytotoxic drugs.** The curves represent the non-linear regression lines calculated for all individual samples included. The dots are individual patient data points and illustrate together with the individual curves the great variability in drug sensitivity between individual samples. Panel **A**: oxaliplatin, **B**: mitomycin C, **C**: 5-fluorouracil, **D**: irinotecan.

**Figure 3:**
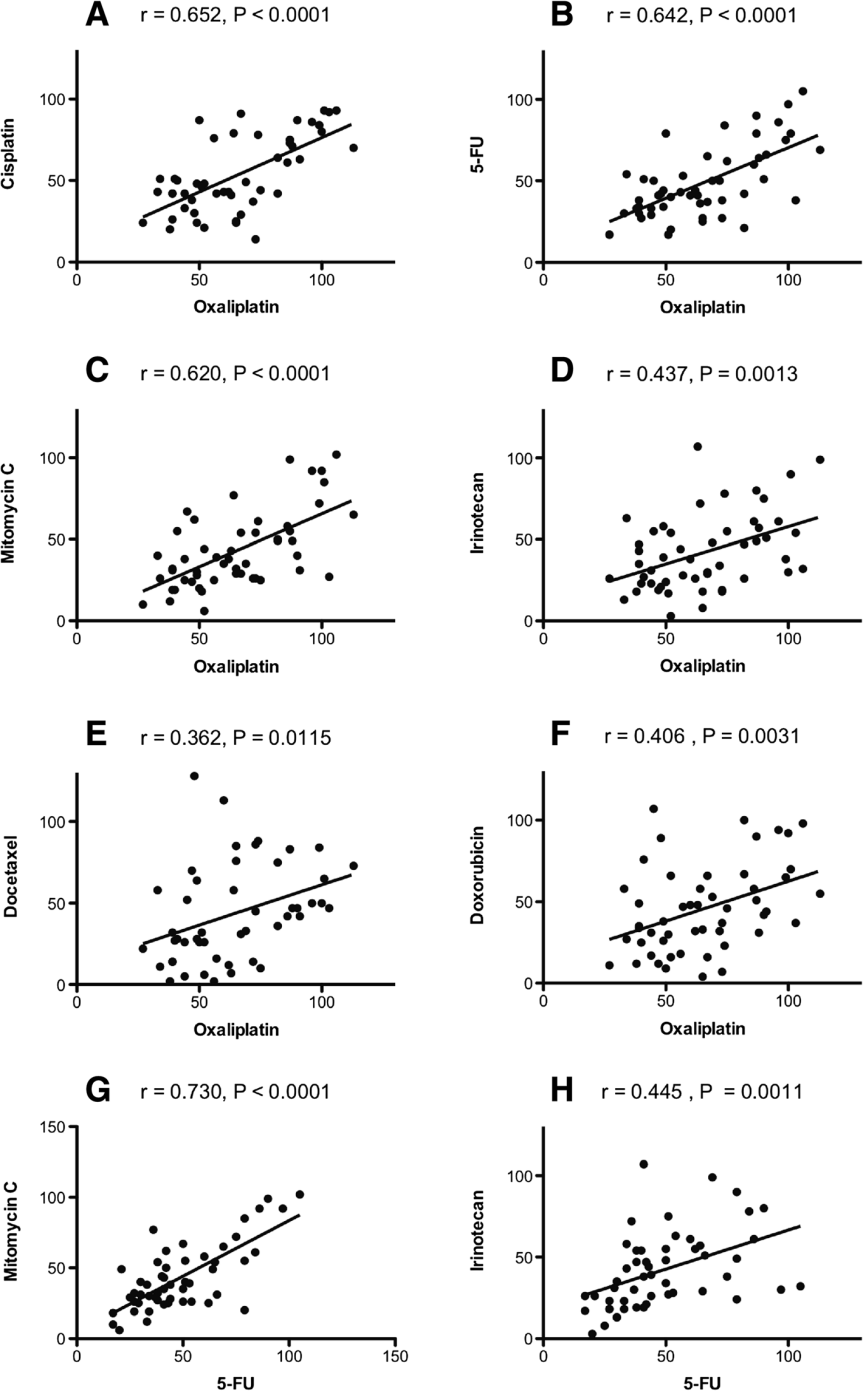
**Correlations between the cytotoxic activities (SI%) for the indicated pairs of standard cytotoxic drugs at concentrations selected to provide optimal activity variation.** The correlations are based on all peritoneal carcinoma samples investigated. The *r* denotes the correlation coefficient and *P* the level of statistical significance. Panel **A**: correlation between cisplatin and oxaliplatin, **B**: 5-fluorouracil and oxaliplatin, **C**: mitomycin C and oxaliplatin, **D**: irinotecan and oxaliplatin, **E**: docetaxel and oxaliplatin, **F**: doxorubicin and oxaliplatin, **G**: mitomycin C and 5-fluorouracil, **H**: irinotecan and 5-fluorouracil.

Some samples were essentially unaffected by the highest drug concentrations tested whereas tumor cells from other samples showed decreased viability even at the lowest concentration tested (Figure 2). The cross-resistance between the different standard drugs investigated was modest to high (Figure 3). In general this means that resistance to one drug would also imply resistance to other drugs. On the other hand, there are clearly many individual samples being resistant to one but sensitive to the other drug, theoretically supporting an individual choice of drugs for the IPC.

### Ex vivo drug sensitivity versus clinicopathological factors for the colorectal cancer subgroup

Table 4 details the mean IC_50_ values of each drug according to different histopathological categories in the CRC subgroup. The mucinous tumors generally had higher IC_50_ values compared to those non-mucinous and this was statistically significant or nearly significant for cisplatin (30 vs. 14 μM, p = 0.05) and irinotecan (184 vs. 81 μM, p = 0.07). Lymph node status at initial diagnosis, tumor grade or signet ring cell type cancer did not consistently affect drug sensitivity. There were essentially no differences in drug sensitivity between CRC PC samples from patients previously exposed to cytotoxic drugs and those being treatment naïve (Table 4).

**Table 4:** **Mean IC**_**50 **_**values of drugs grouped according to histopathological categories and previous treatment in the CRC subgroup**

**Drugs**	**Prior Chemotherapy**		**Lymph node**		**Mucinous**		**Tumour differentiation**			**Signet cell**	
**n=49†**	**Yes**	**No**	**Pos**	**Neg**	**Yes**	**No**	**Poor**	**Moderate**	**Well**	**Yes**	**No**
	**n=39**	**n=10**	**n=37**	**n=12**	**n=29**	**n=20**	**n=19**	**n=24**	**n=6**	**n=6**	**n=43**
Oxa	31.7	30.1 (0.9)	31.0	32.4 (0.9)	36.9	22.6 (0.1)	34.7	23.9 (0.5)	35.3	29.1	31.6 (0.9)
Cis	21.6	26.2 (0.6)	20.1	23.8 (0.9)	29.7	13.6 (0.05)	23.3	17.0 (0.4)	32.8	13.7	24.7 (0.4)
Mel	53.3	52.2 (0.9)	56.1	53.1 (0.8)	52.4	53.8 (0.9)	49.1	53.3 (0.9)	54.8	44.2	52.9 (0.5)
5-FU	539.5	475.4 (0.6)	497.5	604.9 (0.4)	542.1	455.5 (0.4)	521.5	426.6 (0.3)	689.9	490.4	519.2 (0.9)
MMC	19.1	22.5 (0.7)	23.3	12.0 (0.3)	24.6	14.2 (0.2)	12.8	20.5 (0.4)	29.4	8.68	21.2 (0.3)
Iri	131.7	163.1 (0.6)	153.3	111.2 (0.5)	183.9	81.1 (0.07)	122.0	163.5 (0.8)	129.2	84.4	151.6 (0.5)
Doce	15.0	21.5 (0.3)	18.5	13.8 (0.4)	17.2	16.6 (0.9)	16.9	17.9 (0.8)	12.4	23.5	14.4 (0.1)
Dox	2.80	2.98 (0.9)	3.34	1.59 (0.1)	2.96	2.78 (0.9)	1.74	3.51 (0.3)	2.86	1.20	2.96 (0.3)

Numbers in parentheses represent p-values from a Student t-test with the exception of tumor grading which was calculated with a one-way ANOVA.

^†^ Missing histopathological data in 3 patients.

*Abbreviations*: *Pos* positive, *Neg* negative, *Oxa* oxaliplatin, *Cis* cisplatin, *5-FU* 5-fluorouracil, *MMC* mitomycin C, *Iri* irinotecan, *Doce* docetaxel, *Dox* doxorubicin.

Analysis of prognostic impact from histopathology variables and drug sensitivity according to a Kaplan-Meier survival analysis and a multivariable Cox regression model are detailed in Table 5. After adjustment for various prognostic factors in a multivariable model, sensitivity to doxorubicin, synchronous PC and macroscopically radical surgery were independently associated with longer TTP.

**Table 5:** Univariate and multivariable Cox regression model for TTP according to dichotomised IC_50_ drug sensitivity values (above or below the median value) and clinicopathological variables for the CRC subgroup

***n*** **= 47**	**Median TTP**	**Log-rank**	**Univariate**	**Univariate**	**Multivariate**
	**Months**	***p***	**HR**	***p***	***p***
Treated vs. untreated	10 vs. 5	0.06	0.5	0.2	-
Oxaliplatin ^†^	5 vs. 5	0.8	0.9	0.9	-
Cisplatin ^†^	5 vs. 5	0.8	0.9	0.9	-
Melphalan ^†^	12 vs. 7	0.8	1.1	0.8	-
5-FU ^†^	6 vs. 5	0.7	0.9	0.7	-
Mitomycin C ^†^	12 vs. 4	0.2	0.6	0.2	-
Irinotecan ^†^	5 vs. 5	0.9	1.0	0.9	-
Docetaxel ^†^	10 vs. 4	0.6	0.8	0.6	-
Doxorubicin ^†^	12 vs. 1	0.008	0.4	0.009	0.02
Synch. vs. Metach.	9 vs. 4	0.05	0.5	0.05	0.006
Vasc./neural vs. not	2 vs. 10	0.05	2.3	0.03	0.12
Mucinous vs. not	9 vs. 5	0.12	0.6	0.12	-
Lymph node + vs. not	6 vs. 7	0.9	1.0	0.9	-
CC0 vs. CC 1-3	18 vs. 4	0.002	0.3	0.001	0.002

There was missing data concerning TTP in 5 patients.

^†^ Below vs. above the median IC_50_ value. Low IC_50_ indicates better sensitivity.

*Abbreviations*: *TTP* time to progression, *CRC* colorectal cancer, *Synch* synchronous, *metach* metachronous, *vasc/neural* vascular or neural invasion, *CC* completeness of cytoreduction.

**Figure 4:**
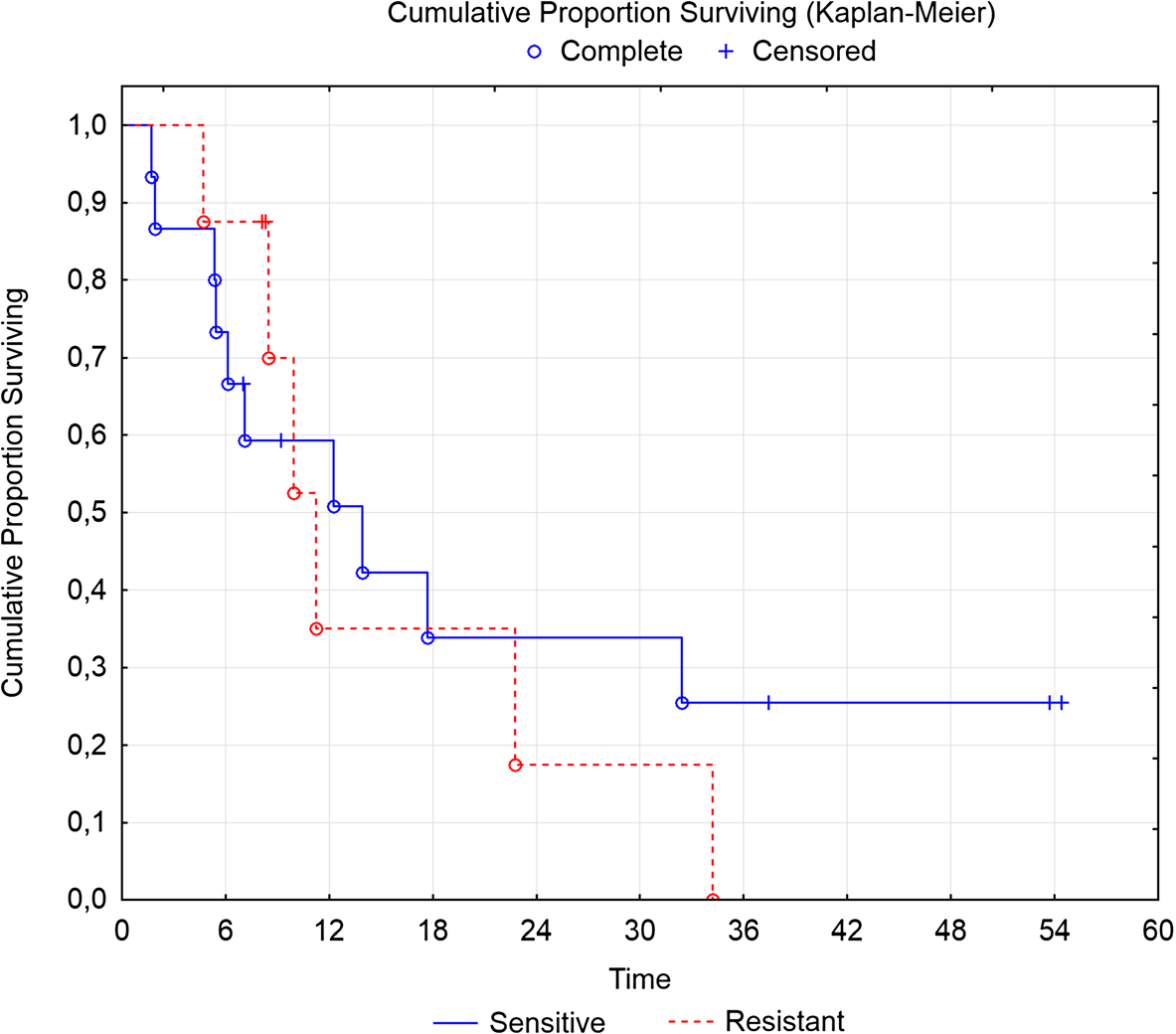
**Kaplan-Meier curve between a more sensitive group and a more resistant group as defined as above or below the median value.** Patients receiving IPC with at least one sensitive drug were included in the sensitive group (*n* = 15) and those receiving only resistant drugs were included in the resistant group (*n* = 8). Only CC 0 patients from the colorectal cancer group were included in the analysis. p = 0.4.

The results of the analysis of the direct relationships between drug sensitivity ex vivo and clinical outcome in individual CRC PC patients are detailed in Figure 4. The Kaplan-Meier curves essentially overlap in the beginning and do not differ statistically significantly. However, at three years, there are three disease free patients in the sensitive group and none in the resistant group. Longer follow-up and more patients are needed for a conclusive analysis.

## Discussion

The FMCA analysis has previously been shown to predict drug efficacy in the clinic both on the diagnosis level and on the individual patient level in hematological malignancies and ovarian cancer [19,20,26]. Therefore, we expect that the FMCA reports clinically relevant drug sensitivity also in the current investigation. CRC, appendix cancer and PMP were generally more resistant to standard cytotoxic drugs than ovarian cancer and mesothelioma. However, for docetaxel in PMP the IC_50_ value approached that of leukemia. This is an interesting observation as docetaxel also showed the lowest cross-resistance to other drugs.

Mesothelioma was surprisingly relatively sensitive across all drugs tested. Cisplatin, the most common agent both for systemic treatment and IPC in PC from mesothelioma [27], is a good choice based on our findings. This is also in line with the treatment results of CRS and IPC for mesothelioma, where the median survival ranges 34–96 months with 5 year overall survival ranging 33-59%, which is better than systemic chemotherapy results with median survival ranging from 9 to 12.5 months [27]. Furthermore, abdominal mesothelioma treated with cytoreductive surgery and IPC appears to have better survival than CRC according to a recent French study which is in line with the FMCA results of greater drug sensitivity [28]. Notably, however, given that the pattern of oxaliplatin activity is very similar to that of cisplatin and the much more favorable concentration ratio for oxaliplatin, use of this drug for IPC in mesothelioma might produce even better results.

Despite having similar drug resistance patterns as CRC, PMP has better survival than both CRC and mesothelioma. The explanation for this is probably that the tumor biology of PMP is more indolent compared with mesothelioma and CRC regardless of the drug resistance pattern [29].

Appendix cancer was similar to CRC in drug sensitivity. Thus, the current practice of treating these two entities with similar chemotherapeutics seems appropriate. For IPC in PC from CRC and appendix cancer, there are two commonly used drugs, i.e. mitomycin C or oxaliplatin. The results of the current study do not give a clear answer to which drug is best but with mitomycin C one may only reach intraperitoneal concentrations that approximate the tumor cell IC_50_ values whereas with oxaliplatin, one might reach 18 times greater a concentration ip. Thus, oxaliplatin seems to be a more suitable drug for IPC. Interestingly, oxaliplatin appears to have the best C_max_ IP/IC_50_ ratio for the other PC diagnoses as well (Table 3).

The variability in ex vivo drug sensitivity in the CRC subgroup was large, as also observed in a previous study on tumor samples from PC [30], ranging from virtually no to total cell death within the concentration range tested. In principle, this argues, together with the observations of low cross-resistance in individual tumor samples, in favor of individualized choice of drug for IPC. This would require tumor tissue sampling and ex vivo drug sensitivity measurements prior to CRS and IPC. Although this would complicate the logistics, it would be feasible since tumor tissue is accessible by laparoscopy and the assay time is 3 days. However, well designed clinical trials evaluating clinical benefit from ex vivo drug sensitivity testing are unfortunately few, sub-optimally designed and do not allow firm conclusions [31].

In PC from CRC, mucinous tumors were generally less drug sensitive than non-mucinous tumors. This is in line with previous results that mucinous CRC is less responsive to systemic chemotherapy than non-mucinous CRC [32]. Interestingly, oxaliplatin and 5-FU were essentially equally active in chemotherapy naïve and previously treated patients. This may be due to patient selection; i.e. previously treated patients suitable for CRS and IPC are those without drug resistant tumors. Still, there is no support for selection of drugs for IPC based on prior treatment status. The same conclusion holds for lymph node status, tumor grade and presence of signet ring cells.

Doxorubicin drug sensitivity was found to be an independent prognostic factor for TTP. This might well be a chance finding but one may speculate that doxorubicin drug sensitivity could indicate CRC tumor cell susceptibility to cancer drugs or that sensitivity to doxorubicin is associated with less aggressive tumor biology.

To support the conclusions and proposals above on the implications of our findings for the use of IPC for PC in the clinic and the role of ex vivo drug sensitivity testing, one would like to know, firstly, that IPC adds benefit to CRS and, secondly, that the ex vivo drug sensitivity data correlates to clinical outcome in patients undergoing IPC. Unfortunately, there is insufficient support for both of these aspects. Support for the benefit from the IPC when added to CRS only derives from one randomized trial in PC from gastric cancer [9], randomized trials in ovarian cancer [8], and from a rodent CRC PC model [11]. In PC from CRC, there was one randomized trial started to elucidate this issue but stopped prematurely after inclusion of 35 patients due to poor patient accrual [33]. There was no trend for benefit from IPC. There is, thus, need for a controlled trial to elucidate this important issue. One such study for CRC (Prodige 7) in France has soon completed its recruitment process.

In the CRC subset, the analysis of the relationship between ex vivo activity of the drugs given to the patient and the clinical outcome in terms of TTP showed no difference between 'sensitive’ and 'resistant’ patients in the short term; but unfortunately, the analysis suffers from major methodological problems related to the limited and heterogeneous data available making the results essentially inconclusive. The number of analyzable patients was a mere 23 (15 vs. 8, Figure 4) as the remaining patients were either open-and-close patients or did not reach complete cytoreduction during the surgery. Furthermore, heterogeneity of drug regimens administered compounded the problem of predictive analysis. A greater number of patients with sufficient follow-up are needed to make a reasonable ex vivo – clinical outcome analysis, which is of importance considering the possible long term effect indicated in Figure 4.

On the other hand, the lack of relationship between the ex vivo drug sensitivity and clinical outcome might be true. This could be the case if the FMCA does not report clinically relevant information. The assay differs from the in vivo situation in several aspects related to both pharmacokinetics and pharmacodynamics. Also relevant, is the dwell time that is significantly longer than in the IPC situation. This may have implications for cell-cycle dependant drugs. However, these differences are more or less inherent in all disease and treatment models, and this will reasonably mean that an ex vivo test like the FMCA will never show a perfect correlation to the clinical outcome. Other reasons for the possible lack of relationship between drug sensitivity and clinical outcome might be that IPC adds no benefit at all to CRS or, alternatively, that the drug concentrations reached during IPC are sufficiently high to overcome drug resistance. Taken together, there are a number of important issues left to investigate when it comes to adding IPC to CRS for treatment of PC.

## Conclusions

Drug-sensitivity varies considerably between PC diagnoses and individual patients arguing for individualized therapy in IPC rather than standard diagnosis-specific therapy. However, in the current paradigm of treatment according to diagnosis, oxaliplatin is seemingly the preferred drug for IPC from a drug sensitivity and concentration perspective. In the CRC subset, analysis of correlation between ex vivo drug sensitivity and TTP was inconclusive due to the heterogeneous nature of the data.

## Abbreviations

IPC: Intraperitoneal chemotherapy; CRS: Cytoreductive surgery; PC: Peritoneal carcinomatosis; PMP: Pseudomyxoma peritonei; CRC: Colorectal cancer; 5-FU: 5-fluorouracil; TTP: Time to progression; HIPEC: Hyperthermic intraperitoneal chemotherapy; MNCs: Mononuclear cells; SPIC: Sequential postoperative intraperitoneal chemotherapy; FMCA: Fluorometric microculture cytotoxicity assay; FDA: Fluorescein diacetate; CC: Completeness of cytoreduction; IC50: 50% inhibitory concentration.

## Competing interests

We, the authors, have no financial or non-financial competing interests.

## Authors’ contributions

PC, HM, WG and PN participated in the study concept and design and in data collection or provision of subject material. HK, RL, and PN contributed in the area of chemosensitivity testing. All authors participated in the data analysis/interpretation as well as in the article writing/critiquing and have given approval to the final draft.

## Pre-publication history

The pre-publication history for this paper can be accessed here:

http://www.biomedcentral.com/1471-2407/13/435/prepub
